# Radioiodinated Phenylalkyl Malonic Acid Derivatives as pH-Sensitive SPECT Tracers

**DOI:** 10.1371/journal.pone.0038428

**Published:** 2012-06-13

**Authors:** Matthias Bauwens, Marijke De Saint-Hubert, Jan Cleynhens, Laura Brams, Ellen Devos, Felix M. Mottaghy, Alfons Verbruggen

**Affiliations:** 1 Laboratory for Radiopharmacy, Faculty of Pharmaceutical Sciences, Katholieke Universiteit Leuven, Leuven, Belgium; 2 Nuclear Medicine, Katholieke Universiteit Leuven, University Hospital Gasthuisberg, Leuven, Belgium; 3 Nuklearmedizin, Rheinisch-Westfaelische Technische Hochschule (RWTH) Aachen, Aachen, Germany; 4 Nuclear Medicine, Maastricht University Medical Centre (MUMC), Maastricht, The Netherlands; Genentech, United States of America

## Abstract

**Introduction:**

*In vivo* pH imaging has been a field of interest for molecular imaging for many years. This is especially important for determining tumor acidity, an important driving force of tumor invasion and metastasis formation, but also in the process of apoptosis.

**Methods:**

2-(4-[^123^I]iodophenethyl)-2-methylmalonic acid (IPMM), 2-(4-[^123^I]iodophenethyl)-malonic acid (IPM), 2-(4-[^123^I]iodobenzyl)-malonic acid (IBMM) and 4-[^123^I]iodophthalic acid (IP) were radiolabeled via the Cu^+^ isotopic nucleophilic exchange method. All tracers were tested *in vitro* in buffer systems to assess pH driven cell uptake. *In vivo* biodistribution of [^123^I]IPMM and [^123^I]IPM was determined in healthy mice and the pH targeting efficacy *in vivo* of [^123^I]IPM was evaluated in an anti-Fas monoclonal antibody (mAb) apoptosis model. In addition a mouse RIF-1 tumor model was explored in which tumor pH was decreased from 7.0 to 6.5 by means of induction of hyperglycemia in combination with administration of meta-iodobenzylguanidine.

**Results:**

Radiosynthesis resulted in 15–20% for iodo-bromo exchange and 50–60% yield for iodo-iodo exchange while *in vitro* experiments showed a pH-sensitive uptake for all tracers. Shelf-life stability and *in vivo* stability was excellent for all tracers. [^123^I]IPMM and [^123^I]IPM showed a moderately fast predominantly biliary clearance while a high retention was observed in blood. The biodistribution profile of [^123^I]IPM was found to be most favorable in view of pH-specific imaging. [^123^I]IPM showed a clear pH-related uptake pattern in the RIF-1 tumor model.

**Conclusion:**

Iodine-123 labeled malonic acid derivates such as [^123^I]IPM show a clearly pH dependent uptake in tumor cells both *in vitro* and *in vivo* which allows to visualize regional acidosis. However, these compounds are not suitable for detection of apoptosis due to a poor acidosis effect.

## Introduction

In contrast to normal tissue, solid tumors commonly have a high metabolic rate and poor perfusion [1]. The elevated anaerobic glycolysis in tumors frequently results in an extracellular acidification [2], [3], [4]. Due to the poor perfusion, this increased extracellular acidity is not washed away by the blood flow, and an intratumoral acidic pH is created. This process was first described by Warburg and is currently known as ‘the Warburg effect’ [5]. Although various alternative pathways have been proposed, including deficiencies in tumor perfusion, metabolic abnormalities associated with transformation, and an increased capacity for transmembrane pH regulation, that each contribute to the intratumoral acidity, the general principle was shown to hold true. Tumor acidity meanwhile has been linked to increased invasion and metastasis formation, partly through hypoxia inducible factor 1 (HIF-1) but also independently. It can therefore be regarded as a prognostic factor for patient survival [6]. In addition, together with poor perfusion and hypoxia, tumor acidity as a physiological factor has a significant impact on the uptake of chemotherapeutics in cancer cells [7]. Weakly basic chemotherapeutic drugs, such as anthracyclines, anthraquinones and vinca alkaloids, are assumed to accumulate in the (intracellular) acidic sections of the cancer cell. The uptake of these compounds can be diminished if the extracellular pH (pH_e_) is decreased. Conversely, the uptake of acidic chemotherapeutic drugs is likely to increase in conditions of lower pH_e_. Attempts were made to modulate pH_e_ in order to facilitate uptake of chemotherapeutic drugs, unfortunately with only moderate success [8]. For example, Raghunand and Robey both showed that increasing intratumoral pH in a preclinical model by means of simple oral administration of sodium bicarbonate led to a decreased number of metastasis [9], [10], [11]. On the other hand, Jahde et al. showed in 1992 that a combination of hyperglycemia and a single administration of meta-iodobenzylguanidine (MIBG) significantly decreased intratumoral pH_e_ from 6.9 to 6.2 [12]. However, this drastic acidification had no significant impact on cell survival.

Therefore, molecular imaging of tumor acidity may provide information on tumor prognosis but also provide insight in treatment efficacy [13]). This has therefore been of interest for many oncologists, although with only minor translation into the clinic so far. Several magnetic resonance imaging (MRI)-based methods for estimation of pH_e_ in animal tumor models have been introduced in recent years. These approaches include the use of T_1_-weighted MRI with contrast agents that exhibit pH-dependent ^1^H relaxation enhancement, ^1^H and ^31^P pH-dependent chemical shift measurements, and ^13^C chemical shift imaging of hyperpolarized ^13^C-labeled bicarbonate [14], [15], [16], [17], [18]. At present, however, these techniques cannot be readily translated to the clinic. In the early eighties of last century ^11^C-labeled tracers were developed for PET that relied on ion trapping to visualize brain tumors as well as brain pH in general. It was found that solid brain tumors were not significantly more acidic than the healthy brain, so could not be readily visualized [19], [20], [21]. More recently, Vavere et al. developed a pH-sensitive probe that showed high uptake in LNCaP and PC-3 tumors, with higher uptake and retention in the more acidic LNCaP tumors [7]. This uptake correlated with differences in the bulk pH_e_ of PC-3 and LNCaP tumors measured in magnetic resonance spectroscopy experiments.

In a different approach, ^18^F-labeled 2-(5-fluoropentyl)-2-methylmalonic acid (^18^F-ML-10) was recently developed as an apoptosis tracer [22], [23]. The rationale is that, when undergoing apoptosis, cells drastically lower their pH_e_, resulting in a specific uptake of ^18^F-ML-10 in apoptotic cells by cell membrane penetration, allowing to visualize apoptosis *in vivo*. The tracer has excellent imaging qualities as it is cleared very rapidly from the body, however its sensitivity for apoptosis imaging *in vivo* has to be elucidated further since only low absolute uptake in apoptotic cells is observed. It may be assumed that cell membrane penetration can be facilitated by making the moderately lipophilic tail on the malonic acid moiety of ^18^F-ML-10 even more lipophilic. The malonic acid moiety itself remains the same, leaving the highest pK_a_ of such a compound roughly identical (pK_a_  = 5.78±0.05).

**Figure 1:**
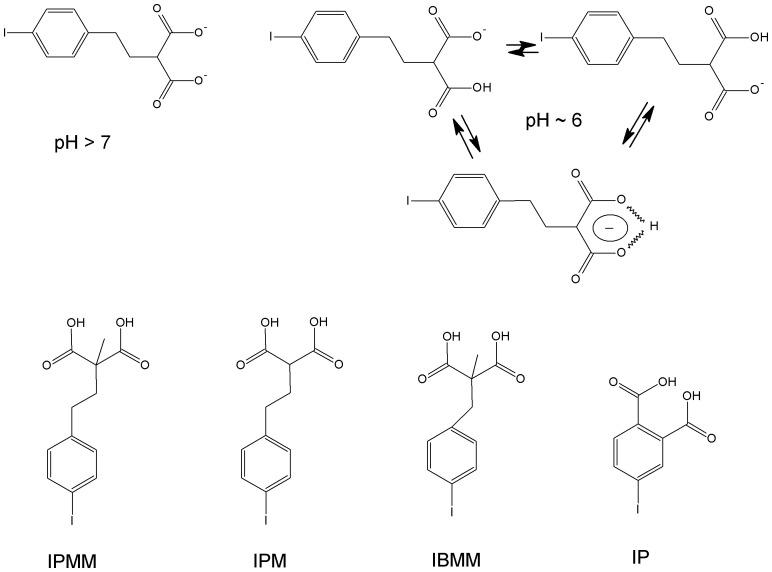
Charge distribution of 2-(4-iodo-phenethyl)-malonic acid (IPM) at pH 7 (2 negative charges) and at pH 6 (the compound contains only one negative charge, which is distributed over the malonate moiety). Given that the highest pK_a_ value for IPM is 5.78, a significant portion of the compound is in this state at pH 6, allowing passive diffusion over a cell membrane. The lower part of the figure depicts the structure of 2-(4-iodo-phenethyl)-malonic acid (IPM), 2-(4-iodo-phenethyl)-2-methyl-malonic acid (IPMM), 2-(4-iodo-benzyl)-2-methyl-malonic acid (IBMM) and 4-iodo-phtalic acid (IP).

In this study, we designed several radiolabeled lipophilic malonic acid derivates and evaluated these as pH-sensitive tracers for SPECT imaging. Figure 1 shows the compounds we evaluated as well as the charge distribution of one of the investigated compounds at pH 6 and 7. To our knowledge, this is the first paper that describes the synthesis, radiosynthesis and pH-sensitivity of iodinated compounds suitable for SPECT.

## Materials and Methods

All chemicals used were of analytical or pharmaceutical grade and were purchased from Sigma-Aldrich, Acros Organics or Fluka. Analytical grade 2-(4-bromobenzyl)-2-methylmalonic acid (BrBMM) and 4-bromo-phthalic acid were purchased from AB Chem and TCI Europe, respectively. For radiolabeling studies sodium [^123^I]iodide in 10^−2^ M NaOH (GE Healthcare) was used.

Identification of the synthesized products was achieved with time-of-flight mass spectrometry (TOF-MS) (LCT, Micromass), with an orthogonal electrospray ionization (ESI) interface. Registration and analysis of the spectra were performed with Masslynx software (version 3.5, Micromass). Radioactivity was measured using an ionization chamber based activity meter (Capintec Radioisotope Calibrator CRC-721) or an automated NaI(Tl) gamma counter (Wallac Wizard). HPLC analyses were performed using an LC apparatus that consisted of a solvent pump (Hitachi Lachrom Elite L-2130 quaternary gradient pump, VWR), a Platinum EPS-C18 column (5 µm, 10 mm×150 mm) (Grace-Alltech), a UV detector (Hitachi L-2400 UV detector) at 254 nm and a 3-inch radiometric NaI(Tl) detector in series.

Mobile phases at a flow rate of 3 ml/min: *Method 1:* 0.1% trifluoroacetic acid (TFA) in water for 3 min, then linear gradient to 0.1% TFA in acetonitrile (ACN) over the course of 17 minutes. *Method 2*: 0.1% TFA in water (A) for 3 min, then linear gradient to 100% ethanol over 17 min.

^1^H-NMR spectra were recorded on a Bruker 400 MHz spectrometer. Chemical shifts are reported in parts per million (ppm) relative to tetramethylsilane (δ  = 0). Coupling constants are reported in hertz (Hz). Splitting patterns are defined by s (singlet), d (doublet), t (triplet) and m (multiplet).

### Synthesis of Precursors

#### 2-(4-Iodophenyl)ethanol (1)

2-(4-Iodophenyl)ethanol was prepared as described in the literature [24].

#### 1-Iodo-4-(2-bromoethyl)benzene (2)

1-Iodo-4-(2-bromoethyl)benzene was also synthesized following a described procedure [25].

#### 2-(4-Iodophenethyl)-malonic acid diethyl ester (3)

To a solution of sodium (0.116 g, 5.06 mmol) in EtOH (5 ml) was slowly added diethyl malonate (9.7 mmol, 1.56 g) and **(2)** (0.746 g, 2.4 mmol) and the mixture was refluxed overnight. After evaporation of the solvent under reduced pressure, the residue was suspended in water and the mixture extracted with EtOAc (2 x 50 ml). The organic layer was washed with 0.5 M HCl (25 ml) and brine (25 ml) and dried over MgSO_4_. After evaporation of the solvent under reduced pressure, the residual yellow oil was purified using silica gel column chromatography with heptane:EtOAc (20∶1, v/v) as eluent. The fractions containing pure **3** were collected and evaporated, yielding 2.08 g (89%) of the desired compound MS (ES)+: m/z [C_15_H_18_IO_4_Na]^+^: calculated: 413 Da; measured: 413 Da. ^1^H-NMR (CDCl_3_): δ 1.26 (6H, t, OCH_2_ *CH_3_*), 2.17(2H, q, CH_2_ *CH_2_*CH); 2.61 (2H, t, *CH_2_*CH_2_CH); 3.30(1H, t, CH_2_ *CH*), 4.19(2H, q, O*CH_2_*CH_3_); 6,92 (2H, d, Ar*_H_*); 7,36 (2H, d, Ar*_H_*).

#### 2-(4-Iodo-phenethyl) malonic acid (IPM, 4)

A solution of compound ***3*** (390 mg, 1 mmol) in a mixture of EtOH:H_2_O (1∶1, v/v) was refluxed for 30 minutes. The solvent was evaporated under reduced pressure and 1 M HCl was added to adjust the pH to 1.5. The mixture was stirred for 30 min at room temperature (RT) and the formed precipitate filtered off, washed with water and dried in a vacuum oven at 60°C to yield 225 mg (58%) of a white product. MS (ES)^-^: m/z [C_15_H_19_IO_4_]^-^: calculated: 332 Da; measured: 332 Da. ^1^H-NMR (CDCl_3_): δ 2,78 (2H, t, *CH_2_*CH_2_OH); 3,80 (2H, t, CH_2_ *CH_2_*OH); 6,96 (2H, d, Ar*_H_*); 7,61 (2H, d, Ar*_H_*). HPLC analysis using method 1 showed a single peak with a R*_t_* of 14.2 min.

#### 2-(4-Iodophenethyl)-2-methyl-malonic acid (IPMM) (6)

The synthesis of IPMM was analogous to that of IPM, starting from 2-methyl malonic acid diethyl ester instead of malonic acid diethyl ester, resulting in 2-(4-iodophenethyl)-2-methyl-malonic acid **(6)**. The yield was comparable (slightly lower) when compared to that of IPM. MS (ES)^-^: m/z [C_12_H_12_IO_4_]^-^Calculated: 346 Da; m/z [C_12_H_12_IO_4_]^-^Sample :346 Da. HPLC analysis using method 1 showed a single peak with a R*_t_* of 15.2 min.

### Radiosynthesis

All radiotracers were prepared using a Cu^+^ assisted labeling method [26], [27]. In a 1-ml septum-closed vial, 28 µl of an aqueous CuSO_4_.5H_2_O solution (1.3×10^−2^ mol/l) was added to a mixture of precursor (1 mg dissolved in 20 µl EtOH), SnSO_4_ (1 mg, 4.5 µmol), gentisic acid (1.25 mg, 6.4 µmol) and citric acid (2.5 mg, 4.7 µmol) in 0.5 ml H_2_O. This solution was flushed with N_2_ for 10 min and 37–185 MBq no carrier added sodium [^123^I]iodide in 10^−2^ M NaOH was added. The reaction vial was heated at 90°C for 60 min and the reaction product was purified using HPLC (method 2). The peak with the purified radiolabeled compound was collected and diluted with 200 µl 0.5 M phosphate buffer (pH 7). The mixture was heated at 70°C under a stream of nitrogen to evaporate the ethanol. The solution was diluted to 1 ml with normal saline and finally passed over a sterile 0.22-µm filter.

Different reaction conditions were tested. Precursor concentration was varied (0.5, 1 and 2 mg) as well as reaction time (10, 20, 40, 50, 60, 70, 80 and 90 min). Reaction temperature was kept constant at 90°C.

### *In vitro* Experiments

To study pH driven uptake of the iodine-123 labeled tracers, their uptake in cells dying through apoptosis or necrosis and in cells in a potassium-rich medium was determined as previously described [28]. In short, apoptosis was induced by addition to the medium of monoclonal anti-Fas antibody, while necrosis was induced by submitting the cells to three freeze-thaw cycles. In addition, the uptake of [^123^I]IPM was evaluated in phosphate buffered solutions, in either saline solution (140 mM NaCl) or a potassium solution (140 mM KCl instead of NaCl), where the pH was varied (ranging from 5.5 to 7.5 in 0.5-unit increments). Tracer uptake was plotted against the in situ pH value.

### *In vivo* Stability, Biodistribution and pH-dependency

All animal experiments were conducted according to the Belgian code of practice for the care and use of animals, after approval from the KULeuven university ethics committee for animals. Shelf-life stability of the radiolabeled compounds was studied with RP-HPLC, using method 2. After sample preparation, *in vitro* and *in vivo* plasma stability was also studied by RP-HPLC. Sample preparation consisted of protein precipitation by addition of acetonitrile and subsequent filtration. The biodistribution of radioiodinated IPMM and IPM was studied in healthy NMRI mice. Radiolabeled IBMM and IP were not further investigated at this point, due to their poor radiolabeling yields. Male NMRI mice were injected intravenously (*i.v.*) under isoflurane anesthesia with 370 kBq of radiolabeled compound (n ≥3 per compound). SPECT imaging (E-cam, Siemens Medical Systems) was performed in healthy mice 45–80 min p.i. of 15 MBq [^123^I]IPM, while CT images were acquired using a Hirez CT scanner (Siemens). In a first attempt to study the pH-dependent uptake of [^123^I]IPM, NMRI mice were administered intravenously 0.1 µg/g (group 1) or 0.2 µg/g (group 2) anti-Fas monoclonal antibody (mAb; Jo2, BD Pharmingen), 90 min prior to *i.v.* administration of [^123^I]IPM. The anti-Fas mAb induces massive hepatic apoptosis, as well as minor levels of apoptosis in the lungs and the spleen [29]. After sacrifice of the animals by decapitation under anesthesia at 60min p.i. of the radiotracer parts of the liver were fixed in 6% formaldehyde for paraffin embedding and histological studies. Five-micrometer sections were cut from paraffin embedded livers for histological staining.

In a second experiment, the uptake of [^123^I]IPM in RIF-1 tumor bearing syngeneic C3H/HeNCrl mice was determined in two conditions. The uptake was first determined in healthy untreated tumor-bearing animals. Secondly, the tumor uptake was studied in animals that were treated with a cocktail of glucose (two injections at 5 min and 60 min prior to tracer injection, 3 mg/g body weight, *i.p.*,) and MIBG (30 µg/g body weight, *i.p.*, 60 min prior to tracer injection) to lower intratumoral pH. A micro pH electrode (Lazar Research Laboratories, Los Angeles, USA) was used to monitor intratumoral pH.

In all experiments the mice were sacrificed by an overdose of Nembutal 60 min post injection (p.i.) of the tracer. The activity of the weighed organs was measured to calculate the percentage of ID (% ID) and % ID/organ weight (%ID/g). Part of the healthy liver, the apoptotic liver and tumor tissue was frozen for autoradiography as previously described (20) and the other part fixed in 6% formaldehyde for paraffin embedding. From paraffin embedded tissues 5-µm sections were cut for standard hematoxylin and eosin (H&E) staining and caspase-3 staining.

### Data Analysis

Image analysis was performed with PMOD software (version 3.1; PMOD Inc.). All data are expressed as mean ± SEM, with n  = 3 or more unless mentioned otherwise. GraphPad Prism (GraphPad) was used for statistical analysis of group results using ANOVA, as well as determining goodness of fit (r^2^, using Spearman rank test).

## Results

### Synthesis and Radiosynthesis

The precursor compounds were synthesized in a moderate but sufficient yield. Their identity was confirmed by MS and ^1^H-NMR.

**Figure 2:**
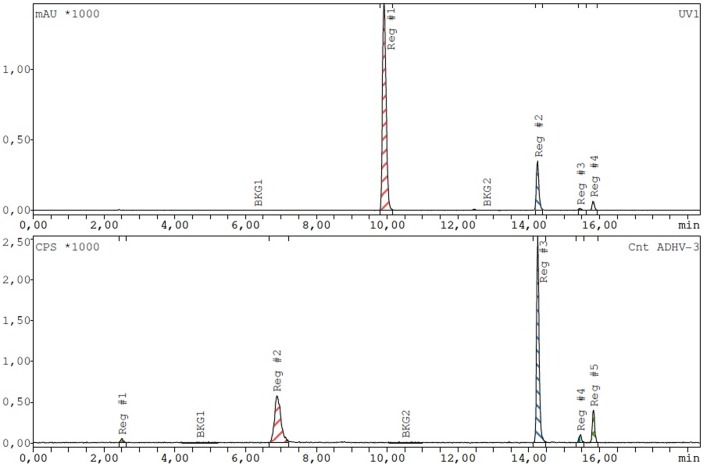
Exemplary UV- (top) and radiochromatogram (bottom) of reaction mixture after radioiodination of IPMM (60 min reaction time, 90°C, 1 mg precursor). In the UV signal, gentisic acid (Rt: 10 min), IPMM (Rt: 15 min) and IPMM diethyl ester (R_t_: 16 min) can be identified. In the radiochromatogram, ^123^I-labeled IPMM and ^123^I-labeled IPMM diethyl ester can be seen, as well as an unidentified side compound (R_t_: 7 min).

Radioiodination of 2-(4-bromobenzyl)-2-methylmalonic acid and 4-bromophthalic acid by nucleophilic exchange resulted in a maximal yield of 15–20%. IPM and IPMM were radioiodinated by isotopic nucleophilic exchange with a maximal yield of 60%. Precursor amount (in the range 0.5–2 mg) did not have impact on this result, and a maximal yield was obtained after 40 min reaction time at 90°C. The remaining 40% of the radioactivity was associated with an unidentified side product that was quickly formed (within 10 min of heating), but easily separated from ^123^I-labeled IPM or IPMM on HPLC (figure 2). The percentage of iodine-123 in the form of iodide was below 3% in optimal labeling conditions. HPLC purification allowed for the collection of pure ^123^I-labeled IBMM, IP, IPM and IPMM (retention times respectively 14.5, 13.5, 14.5 and 15.0 min) in a volume of maximum 3 ml (1 min collection). Separation of brominated from iodinated compound was not attempted. After addition of 200 µl 0.5 M phosphate buffer (pH 7), subsequent evaporation did not affect compound stability and allowed to obtain the pure compound in an ethanol-free solution.

### *In vitro* Evaluation

**Figure 3:**
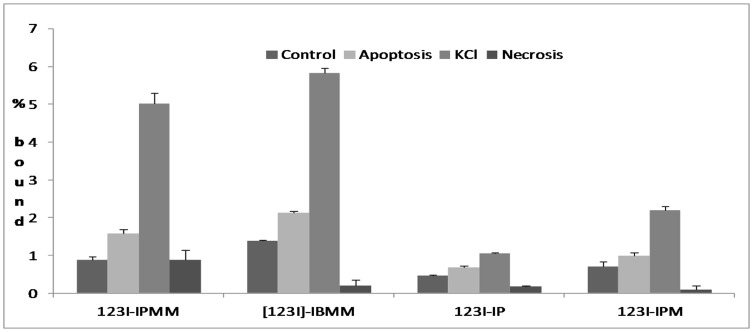
Uptake of ^123^I-labeled IPMM, IBMM, IP and IPM in Jurkat cells in control conditions, with anti-fas mAb induced apoptosis, incubated in KCl-solution and with freeze-thaw induced necrosis.

Figure 3 shows the uptake of ^123^I-labeled IPMM, IBMM, IP and IPM in different conditions. Increased uptake of all tracers was observed in apoptotic cells and cells incubated in KCl-phosphate buffer (PB) medium compared to NaCl-phosphate buffer control conditions. In comparison to apoptotic cells, cells in KCl-PB showed the most pronounced uptake. No increased uptake was observed in cells undergoing necrosis. No significant in situ pH decrease was noticed in any of the conditions, except for cells in a KCl-PB medium where pH decreased by about 0.5 pH units.

**Figure 4:**
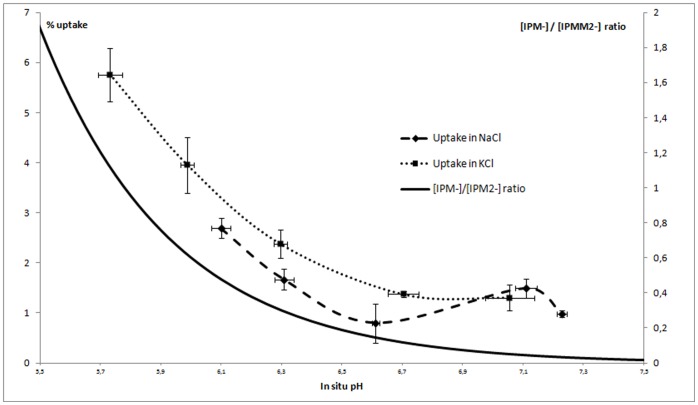
Uptake of ^123^I-labeled IPM in Jurkat cells after 3 h incubation in phosphate buffered NaCl or phosphate buffered KCl solutions as a function of in situ pH. The [IPM^−/^[IPM^2−^] ratio is shown for comparison (dotted line).

Although the absolute uptake of the tracer showed significant differences, the ratios of uptake in apoptotic cells versus control cells ranged from 1.4 (for 4-iodophthalic acid) to 1.8 (for IPMM). The ratios of uptake in KCl-PB incubated cells vs. cells in control conditions ranged from 2.3 (for IP) to 5.7 (for IPMM). The effect of KCl on the uptake of [^123^I]IPM as a function of pH is illustrated in figure 4. It is clear that the KCl-PB solution lead to more acidic in situ pH values, correlating with an increased uptake of ^123^I-labeled IPM at pH values below 6.5. However, even at identical in situ pH, the uptake of [^123^I]IPM in a KCl-PB was higher compared to that in a NaCl-PB, indicating that in addition to the pH effect KCl also increased tracer uptake via another pathway, namely the described plasma membrane depolarization [23]. The uptake of [^123^I]IPM in Jurkat cells was linearly correlated with the ratio of [IPM^-^]/[IPM^2−^] (r^2^>0.95).

### *In vivo* Evaluation

**Figure 5:**
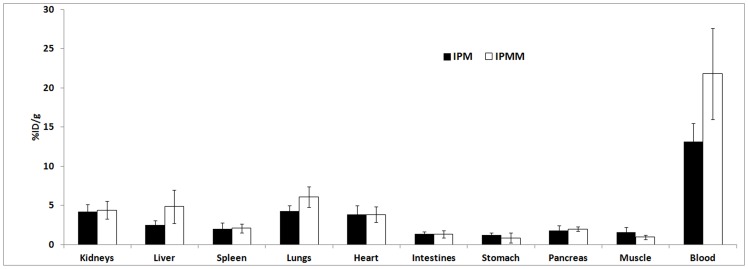
Biodistribution of ^123^I-labeled IPMM and IPM in healthy NMRI mice, 60 min p.i.

**Figure 6:**
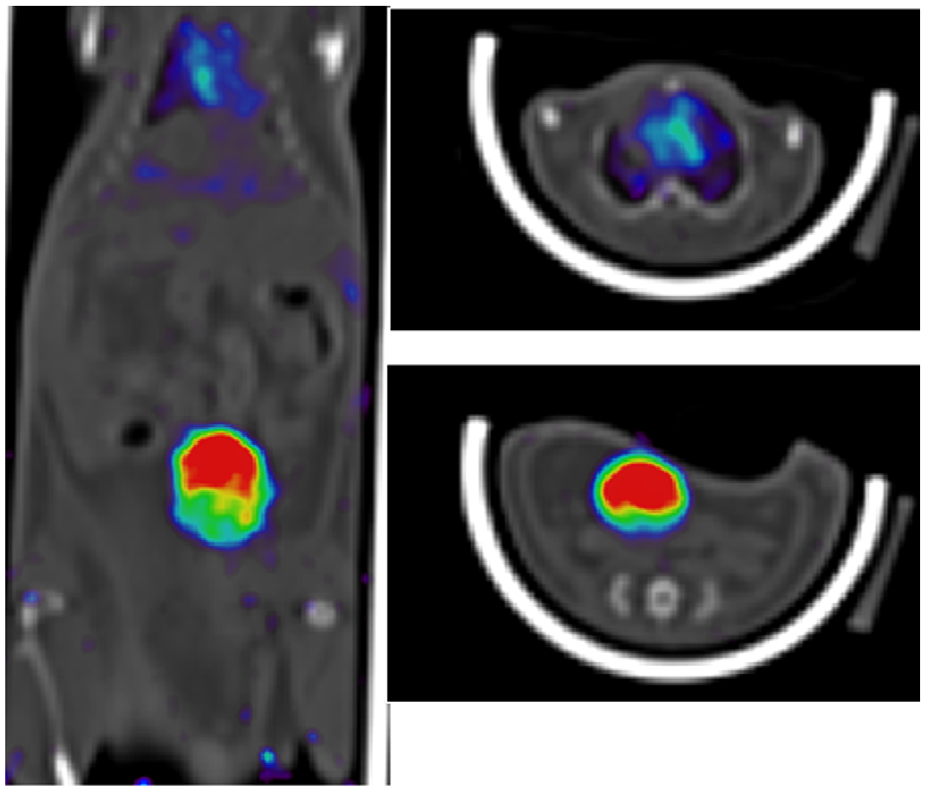
SPECT/CT image of [^123^I]-labeled IPM in healthy NMRI mice, acquired between 45–80 min p.i. (summed image). (A) Coronal section of the mouse body (head not included), showing high uptake in bladder and heart (blood pool). (B) Transversal section of the mouse in the heart region. (C) Transversal section showing bladder activity.

After *i.v.* injection of ^123^I-labeled IPMM and IPM in healthy mice, analysis of plasma and urine samples at 60 min p.i. did not reveal any radioactive metabolites as shown by HPLC analysis. The biodistribution of ^123^I-labeled IPMM and IPM in healthy mice at 60 min p.i. is shown in figure 5. Both compounds are slowly cleared renally (respectively 33.6±9.9 and 19.0±7.1%ID in the urine at 60 min p.i.). The biodistribution pattern is roughly similar for both compounds, including a high blood pool retention and high activity in all highly perfused organs. Figure 6 shows a SPECT/CT image of the biodistribution of [^123^I]IPM in a NMRI mouse acquired between 45–80 minutes p.i. The activity in the bladder and the blood pool can easily be observed, while the tracer is uniformly distributed.

In the anti-Fas mAb treated mice, the degree of apoptosis was strongly dependent on the administered dose of anti-Fas mAb: 15–30% of hepatic cells were apoptotic in the group that received 0.1 µg/g, while 85–95% of hepatic cells were apoptotic in the group that received 0.2 µg/g. [^123^I]IPM hepatic uptake in anti-Fas mAb treated mice, when compared to hepatic uptake in control conditions, did not increase in the 0.1 µg/g group but increased in the 0.2 µg/g group, although not significantly (p  = 0.18). There was no linear correlation between the degree of hepatic apoptosis and hepatic uptake of [^123^I]IPM (r^2^ = 0.476). No pH decrease was noticed in the liver of any of the models. No increased uptake in the spleen or lungs was noticed for any tracer.

**Figure 7:**
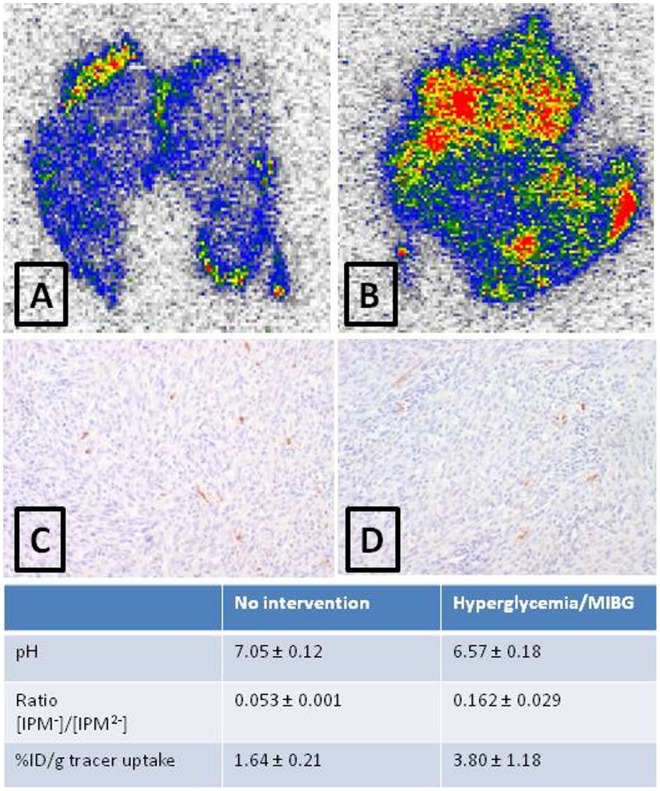
pH dependency of tumor uptake. Autoradiographic images of tumor slices from untreated (A) and treated (B) (hyperglycemia + MIBG) mice. (C) and (D) are caspase-3 stainings of respective tumor tissues. The table indicates pH, [IPM^-^]/[IPM^2−^] ratio at indicated pH and the uptake of the tracer, expressed as %ID/g.

The biodistribution of [^123^I]IPM in RIF-1 tumor bearing C3H/Henkel mice was similar to that in NMRI mice, with tumor uptake in normal conditions amounting to 1.64±0.21%ID/g. Glycemia was 207±57 mg/dl in these animals and intratumoral pH was 7.05±0.12. In mice treated with a cocktail of glucose and MIBG glycemia increased to 356±139 mg/dL and tumor pH decreased to 6.57±0.18, whereas pH of liver and muscle remained unchanged. No increase in the extent of apoptotic cells in the tumor was noticed. The uptake of [^123^I]IPM in the more acidic tumor was 3.80±1.18 times higher than in the tumor of untreated animals (p  = 0.003). Autoradiography (figure 7) confirmed a 3 times higher uptake in the more acidic tumor and showed regional hot spots of activity in the tumor tissue.

## Discussion

The physiological differences between normal and diseased tissues, especially tumor tissues, provide an opportunity for development of specific diagnostic tracers. Especially the pH of tumor tissue, if acidic, provides a strong prognostic factor for metastasis formation and thus patient survival. However, the intratumoral acidic environment has up to now not been adequately exploited for imaging purposes. So far, few compounds that show significant changes in properties, *e.g.* charge, in the pH range between 6.0 and 7.5 have been developed and no clinical data are available. Vavere et al. developed a ^64^Cu-labeled pH-sensitive peptide that showed high uptake in LNCaP and PC-3 tumors, with higher uptake and retention in the more acidic LNCaP tumors [7]. This compound is very promising and further research is necessary to validate the tracer.

Inspired by the use of a ^18^F-labeled malonic acid analogue for PET imaging of apoptosis *in vivo* [23], we developed in this study new lipophilic radiolabeled malonic acid analogues for imaging pH differences using SPECT. Malonic acid has pK_a_ values of 2.83 and 5.69, while 2-methylmalonic acid has pK_a_ values of 3.07 and 5.78. This means that, as soon as the physiological pH is below 6.5, a significant proportion of the compound has a net charge of −1, which is shared throughout the malonic acid moiety by a hydrogen-bridge between the two carboxyl functions [23].

In these conditions, malonic acid and its derivatives are lipophilic enough to be transported through the cell membrane. Once inside, the higher intracellular pH further deprotonates the malonic acid moiety, resulting in a net charge of −2, rendering the compound unable to rediffuse across the membrane.

We successfully synthesized IPMM and IPM. Radiosynthesis of ^123^I-labeled IPMM and IPM using Cu^+^ catalyzed isotopic exchange was optimized, with a final yield of 60%. Radiosynthesis of ^123^I-labeled IBMM and IP, starting from brominated precursors, was achieved with a yield of 15–20%. Considering the better properties of an iodo substituent as a leaving group, such yields are in the range of expectance. Unfortunately, radioiodine for iodine substitution does not allow radiosynthesis with a high specific activity (expressed in MBq/mmol compound). Although specific activity may not be important in pH-driven membrane crossing, it may be advantageous to use electrophilic substitution using a tributyl or trimethyl stannyl leaving group for future studies as they do allow easy separation of precursor and radiolabeled compound.

The data from the *in vitro* experiments clearly demonstrate that all four tracers show a higher uptake in an acidic environment. The acidification caused by apoptosis *in vitro* is limited (decrease of <0.1 pH unit), causing only a 1.4–1.8 fold increase in tracer uptake. On the other hand the stronger acidification and plasma membrane depolarization caused by KCl (>0.5 pH unit) resulted in a 2.3–5.7 fold increase in tracer uptake. More detailed analysis showed that it is mainly the acidification that causes increased tracer uptake. [^123^I]-IP showed the lowest increase in pH-related uptake, presumably due to its lower pK_a_ values when compared to 2-methylmalonic acid (5.51 vs 5.78).

Due to the poor labeling yields (expressed as % radiolabeled compound with respect to the starting amount of radioactivity, non-decay corrected) when starting from brominated precursors, only ^123^I-labeled IPMM and IPM were evaluated *in vivo*. Biodistribution studies showed a high blood pool activity and slow clearance for both compounds, with slightly lower blood pool values for [^123^I]IPM. This high blood pool activity probably results from the high lipophilicity of the molecule which could potentially hamper the use for in vivo molecular imaging and must be kept in mind when further developing pH sensitive tracers. Apart from the higher uptake in well perfused organs, no increased uptake was noticed in any specific organ. Anti-Fas mAb induced hepatic apoptosis did not correlate with a higher hepatic uptake of [^123^I]IPM. Most likely the decrease in pH as a result of apoptosis is insufficient to allow a significant uptake of the tracer *in vivo*. Nevertheless the increased hepatic uptake in the 0.2 µg/g group can be related to a small pH decrease as a result of apoptosis although it may also be explained by vascular disruption and leakage due to apoptosis [30]. Taken together with the minor uptake in apoptotic cells *in vitro*, this finding indicates that the malonic acid derivates investigated in this study are not reliable as apoptosis tracers *in vivo*.

Using a cocktail of MIBG and glucose, inducing tumor acidosis by inhibiting the citric acid cycle (MIBG-induced) and increasing glucose consumption (glucose-induced effect), [^123^I]IPM showed a 2.5-fold higher uptake in acidic tumors (pH 6.57) as compared to normal tumors (pH 7.05). It can be assumed that acid derivatives with a higher pK_a_ value, such as maleic acid (pK_a_  = 6.07) may be more sensitive and show an even higher uptake in acidic tumors.In any case, pre-treatment of subjects with MIBG is recommended to intensify tumor uptake. MIBG has been used safely in the past, in experimental studies as well as in clinical practice, as an attempt to treat tumors by acidosis induction [12] but also as a radiolabeled agent for molecular imaging (labeled with ^123^I) or even treatment (labeled with ^131^I) of neuroblastoma [31], [32], [33].

Further studies are required to validate the usefulness of [^123^I]IPM, preferably comparing the uptake in metastasis forming acidic tumors to that in lower grade tumors.

### Conclusion

An early evaluation of cancer therapy by means of apoptosis imaging is not possible with [^123^I]IPM due to the low pH decrease in apoptotic tissue.

^123^I-labeled malonic acid derivates, such as [^123^I]IPM, do show a clear pH dependent uptake in tumor cells both *in vitro* and *in vivo* and can visualize regions of acidosis. If this can be applied in a clinical setting, this would allow a vast improvement in the assessment of tumor progression due to the strong link between tumor acidosis and metastasis formation. If the sensitivity of [^123^I]IPM is further confirmed, this could make pH-sensitive SPECT imaging a new factor in patient diagnosis, prognosis and treatment planning.
